# The A^2^DS^2^ Score as a Predictor of Pneumonia and In-Hospital Death after Acute Ischemic Stroke in Chinese Populations

**DOI:** 10.1371/journal.pone.0150298

**Published:** 2016-03-07

**Authors:** Xiaopei Zhang, Shangzhen Yu, Lin Wei, Richun Ye, Meizhen Lin, Xiaomin Li, Guoming Li, Yefeng Cai, Min Zhao

**Affiliations:** 1 Department of Neurology, Guangdong Provincial Hospital of Chinese Medicine, Guangzhou, Guangdong, China; 2 Department of Neurology, JiangmenWuyi Traditional Chinese Medicine Hospital, Jiangmen, Guangdong, China; 3 National Clinical Research Center for Kidney Disease, State Key Laboratory for Organ Failure Research, Nanfang Hospital, Southern Medical University, Guangzhou, Guangdong, China; Massachusetts General Hospital, UNITED STATES

## Abstract

**Background and Purpose:**

Stroke-associated pneumonia (SAP) is a common complication and an important cause of death during hospitalization. The A^2^DS^2^ (Age, Atrial fibrillation, Dysphagia, Sex, Stroke Severity) score was developed from the Berlin Stroke Registry and showed good predictive value for predicting SAP. We sought to identify the association between the A^2^DS^2^ score and SAP, and, furthermore, to identify whether the A^2^DS^2^ score was a predictor for in-hospital death after acute ischemic stroke in a Chinese population.

**Methods:**

This was a retrospective study. 1239 acute ischemic stroke patients were classified to low A^2^DS^2^ group (0–4) and high A^2^DS^2^ score (5–10) group. Primary outcome was in-hospital SAP. Logistic regression analyses were performed to identify the association between the A^2^DS^2^ score and SAP, and also the association between the A^2^DS^2^ score and in-hospital death.

**Results:**

The overall incidence rates of SAP and in-hospital mortality after acute ischemic stroke were 7.3% and 2.4%, respectively. The incidence rate of SAP in low and high A^2^DS^2^ score groups was separately 3.3% and 24.7% (P<0.001). During hospitalization, 1.2% patients in low score group and 7.8% patients in high score group died (P<0.001). Multivariate regression demonstrated that patients in high score group had a higher risk of SAP (OR = 8.888, 95%CI: 5.552–14.229) and mortality (OR = 7.833, 95%CI: 3.580–17.137) than patients in low score group.

**Conclusions:**

The A^2^DS^2^ score was a strong predictor for SAP and in-hospital death of Chinese acute ischemic stroke patients. The A^2^DS^2^ score might be a useful tool for the identification of patients with a high risk of SAP and death during hospitalization.

## Introduction

Stroke-associated pneumonia (SAP) is a common medical complication after stroke, with rates reported between 5.6% and 37.98% [1–11]. SAP frequently occurs in the first week after stroke onset, especially the first 3 days [1, 3]. Evidence shows that SAP is an important risk factor for mortality after stroke[12–16]; 19.1–26% of post-stroke patients with pneumonia, but only 3.5–5% of patients without pneumonia[5,14,15] die during hospitalization. Moreover, SAP increases length of stay (LOS) and hospitalization costs [5,7,9,15], which increase burdens on family and society.

Risk factors for SAP include older age[17–23], male[19,24], atrial fibrillation[19–23], stroke severity[17–20,22,23], dysphagia[2,17–20,22] and total anterior circulation infarct (TACI) or posterior circulation infarct (POCI) stroke subtypes[20,22]. To effectively evaluate the risk of SAP, several scales have been developed, including Kwon’s pneumonia score[17], Sellars’s predictive model[2], Chumbler’s 3-level scoring system[18], Ji’s AIS-APS (acute ischemic stroke-associated pneumonia score)[20], and Smith’s ISAN score[23]. However, these scoring systems are not widely used in routine clinical practice due to small sample size[17], lack of validation[17,18], retrospective nature[18], the unavailability or delayed availability for obtaining predictors(e.g., low abbreviated mental test scores, chronic obstructive pulmonary disease, congestive heart failure)[2,20] and the inability to incorporate important risk factors (e.g., dysphagia)[21,23].

The A^2^DS^2^ (Age, Atrial fibrillation, Dysphagia, Sex, Stroke Severity) score is a simple scoring system developed from routinely collected data that was available immediately after hospital admission. It was developed from the Berlin Stroke Registry (BSR) cohort[19]and was subsequently validated using German[19], China[22] and United Kingdom[23]stroke registry data. The previous study used the A^2^DS^2^ score as a continuous variable to predict SAP[22]. However, in the clinical practice, the dichotomized cutoff point was more convenient. Several studies suggested the A^2^DS^2^score^’^s cutoff was 5 point, which represented maximum Youden index[19,20,22].The objective of our study is to identify the association between the A^2^DS^2^ score and SAP, also the association between the A^2^DS^2^ score and in-hospital death in a Chinese acute ischemic stroke population when the A^2^DS^2^ score was dichotomized into low(0–4) and high(5–10) score groups.

## Patients and Methods

### Study Population

Subjects were acute ischemic stroke (AIS) patients admitted to the department of neurology in Guangdong Provincial Hospital of Chinese Medicine between August 2005 and July 2008. Inclusion criteria were: (1) ischemic stroke verified by Computerized Tomography (CT) or Magnetic Resonance Imaging (MRI); and (2) time from symptom onset within7 days. Patients were excluded if any of the components of the A^2^DS^2^ score were not available.

### Ethics Statement

Our study was approved by the ethic committee (2008 GL-37) of 2nd Affiliated Hospital of Guangzhou University of Chinese Medicine. Our study was a retrospective study and all information was pulled out through the electronic system. There was no consent of patients but all the data was analyzed anonymously.

### Data Definitions and Data Extraction

Patients’ records with cerebral infarction or ischemic stroke were obtained from the medical record system. The investigators verified the diagnosis according to the CT/MRI results and excluded patients who had a stroke more than 7 days. Finally, patients who met criteria were enrolled in our study. The following information of the patients were pulled out from the system: (1) demographics (i.e., age and sex); stroke risk factors (i.e., hypertension, diabetes mellitus, dyslipidemia, and atrial fibrillation, including a history of atrial fibrillation or documentation of atrial fibrillation at admission, coronary heart disease (CHD), stroke and TIA history, smoking, and drinking); (2) stroke severity at admission as assessed by the National Institute of Health Stroke Scale score (NIHSS); dysphagia (Kubota’s water swallow test Grade III or higher)[25]; (3)outcomes including pneumonia and death during hospitalization.

The A^2^DS^2^ score was calculated (Age≥75 years = 1, Atrial fibrillation = 1, Dysphagia = 2, male Sex = 1, stroke Severity, National Institutes of Health Stroke Scale 0–4 = 0, 5–15 = 3, ≥16 = 5)[19] and was dichotomized into low (0–4) and high (5–10) score groups[19,20,22].

### Primary and Secondary Outcomes

The primary outcome of our study is SAP and the secondary outcome is death during hospitalization. In this study, SAP was diagnosed based on the original medical documents and re-evaluated by the treating physician according to Mann’s diagnostic criteria[26] for pneumonia based on the presence of ≥3 of the following variables: fever (>38°C), productive cough with purulent sputum, abnormal respiratory examination (tachypnea [>22/min], tachycardia, inspiratory crackles, bronchial breathing), abnormal chest radiograph, arterial hypoxemia (PO_2_<70mmHg), and isolation of a relevant pathogen (positive gram stain and culture).Patients who had pneumonia before their stroke were not included. Pneumonia that occurred within the first 72 hours after the stroke onset was defined as early-onset pneumonia (EOP)[1]. The routine procedure of nursing care of stroke patients in our stroke unit included dysphagia screening, oral cavity cleaning, head-up position, and regular chest care, which were all done persistently to prevent SAP. Prophylactic antibiotics were not used to prevent pneumonia.

### Statistical Analysis

The data were presented as the mean ±standard deviation (SD), the median with interquartile ranges (IQR), or frequencies with percentages, as appropriate. Continuous variables were analyzed with Student’s t- or Kruskal-Wallis tests and categorical variables were analyzed with Chi-square tests. The A^2^DS^2^ score was analyzed as both continuous and binary variable. To estimate crude and adjusted odds ratios of the A^2^DS^2^ score for SAP and in-hospital death, univariate and multivariate logistic regression analyses were performed with SAP and in-hospital death as binary outcome. P values < 0.05 were considered to be statistically significant. In logistic regression analysis, accurate estimation of the discriminant function parameters demands sample size of minimum 20 cases for each predictor variable. All analyses were performed using Empower(R) (www.empowerstats.com, X&Y Solutions, Inc., Boston, MA) and R (http://www.R-project.org).

## Results

### Clinical Characteristics

**Fig 1:**
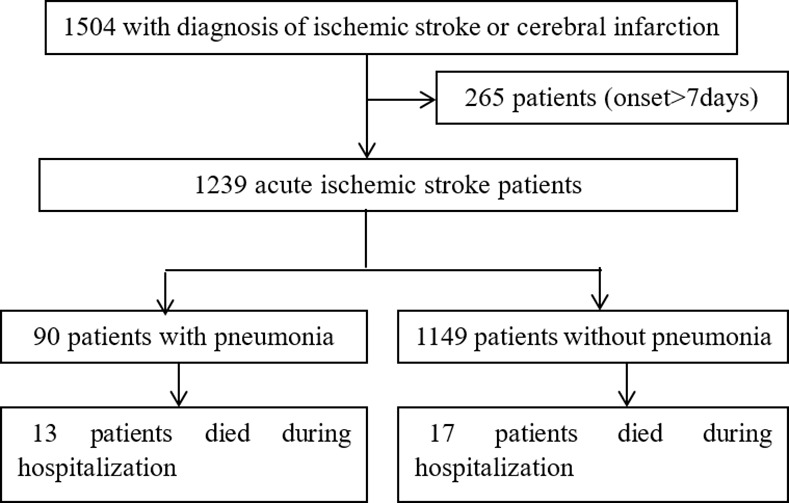
Study population flowchart.

Records of 1504 patients with the diagnosis of “ischemic stroke” or “cerebral infarction” were extracted from the electronic medical system. 265 patients were excluded because time from stroke onset was more than 7 days. At last, 1239 patients were analyzed, among which 90 patients (7.3%) had SAP and 30 patients (2.4%) died during hospitalization (Fig 1).

The patients’ mean age was 69.056±11.662, and 732 patients (59.1%) were men.131 patients (10.6%) had atrial fibrillation, 204 patients (16.5%) had dysphagia symptoms, and the NIHSS median score was 3 (IQR 2–6). The median A^2^DS^2^ score was 2(IQR1-4).1008 patients (81.4%) were in the low A^2^DS^2^ score group and 231(18.6%) were in the high A^2^DS^2^ score groups (Table 1).

**Table 1:** Characteristics of the study population (n = 1239).

Characteristic	Total	SAP(n = 90)	Without SAP (n = 1149)	P value
Age, Mean (SD)	69.056(11.662)	68.574(11.773)	75.211(7.915)	<0.001
Age group, ≥75, n (%)	451(36.4)	47(52.2)	404(35.2)	0.001
Sex (Male), n (%)	732(59.1)	57(63.3)	675(58.7)	0.394
Vascular risk factor, n (%)				
Hypertension	797(64.3)	61(67.8)	736(64.1)	0.478
Diabetes mellitus	233(18.8)	22(24.4)	211(18.4)	0.165
Dyslipidemia	67(5.5)	4(6.0)	63(5.5)	0.680
Atrial fibrillation	131(10.6)	19(21.1)	112(9.7)	0.001
Coronary heart disease	146(11.8)	22(24.4)	124(10.8)	<0.001
Previous stroke /TIA	334(27.0)	32(35.6)	302(26.3)	0.056
Smoking	399(32.2)	32(35.6)	367(31.9)	0.480
Drinking	231(18.6)	20(22.2)	211(18.4)	0.365
Dysphagia, n (%)	204(16.5)	63(70.0)	141(12.3)	<0.001
Admission NIHSS score, Median (IQR)	3(2–6)	6(3–10)	3(2–6)	<0.001
A^2^DS^2^ score, Median (IQR)	2(1–4)	6(3–6)	2(1–4)	<0.001
A^2^DS^2^ score group, n (%)				<0.001
low score group (0–4)	1008(81.4)	33(36.7)	975(84.9)	
high score group (5–10)	231(18.6)	57(63.3)	174(15.1)	

TIA indicates transient ischemic attack; NIHSS, National Institutes of Health Stroke Scale; IQR, interquartile range.

The overall incidence rate of SAP and in-hospital mortality was 7.3% and 2.4%, respectively. The proportion of SAP varied from 0.6% to 41.9% in patients with different A^2^DS^2^ score points. The incidence rate of SAP during the hospitalization in the low and high score groups were 3.3% and 24.7% (P<0.001), respectively. During hospitalization, 1.2% patients in the low and 7.8% patients in the high score group died (P<0.001).

### Risk Factors for SAP

The univariate analysis showed that age, atrial fibrillation, dysphagia, admission NIHSS score, history of CHD, and high A^2^DS^2^ score were risk factors for SAP (Table 2). The further multivariate analysis proved that higher A^2^DS^2^ score was associated with higher risk of SAP (OR = 1.759; 95%CI, 1.560–1.984) even after adjustment for traditional stroke risk factors. Subjects in high A^2^DS^2^ score group had higher risk for SAP (OR = 8.888; 95%CI, 5.552–14.229) compared to low A^2^DS^2^ score group.

**Table 2:** Univariate analyses of factors related to SAP.

Characteristic	SAP, n (%)	OR	95%CI	P Value
Age, ≥75	47(10.4)	2.016	1.310–3.101	0.001
Sex (Male)	57(7.8)	1.213	0.778–1.892	0.395
Atrial fibrillation	19(14.5)	2.478	1.440–4.262	0.001
NIHSS at admission	90(7.3)	1.221	1.155–1.290	<0.001
Dysphagia	63(30.9)	16.681	10.280–27.067	<0.001
Hypertension	71(7.7)	1.180	0.746–1.866	0.478
Diabetes mellitus	22(9.4)	1.438	0.869–2.379	0.157
Dyslipidemia	4(6.0)	0.804	0.286–2.263	0.680
Coronary heart disease	22(15.1)	2.674	1.597–4.479	<0.001
Previous stroke /TIA	32(9.6)	1.547	0.986–2.430	0.058
Smoking	32(8.0)	1.176	0.750–1.842	0.480
Drinking	20(8.7)	1.270	0.756–2.134	0.366
High A^2^DS^2^ score group	57(24.7)	9.68	6.12–15.30	<0.001

SAP indicates stroke-associated pneumonia; TIA, transient ischemic attack; NIHSS, National Institutes of Health Stroke Scale.

### Risk Factors for In-hospital Mortality

The univariate analysis showed that hypertension, atrial fibrillation, dysphagia, NIHSS at admission and the A^2^DS^2^ score were risk factors for death during hospitalization (P<0.05) (Table 3). Multivariate logistic regression showed that the A^2^DS^2^ score’s OR for in-hospital mortality was 1.753 (95%CI, 1.444–2.129) (P<0.001) after adjustment for vascular risk factors. Patients in the high score group had a higher risk of in-hospital death than patients in the low score group (adjusted OR = 7.833; 95% CI, 3.580–17.137) (P<0.001) (Table 4).

**Table 3:** Univariate analyses of factors related to in-hospital death.

Characteristic	Death, n (%)	OR	95%CI	P
Age, ≥75	12(2.7)	1.169	0.558–2.450	0.679
Sex (Male)	19(2.6)	1.202	0.567–2.547	0.632
Atrial fibrillation	10(7.6)	4.496	2.057–9.827	<0.001
Dysphagia	22(10.8)	15.518	6.805–35.389	<0.001
NIHSS at admission	30(2.4)	1.179	1.079–1.288	<0.001
Hypertension	25(3.1)	2.830	1.076–7.446	0.035
Diabetes mellitus	6(2.6)	1.081	0.437–2.677	0.865
Dyslipidemia	4(6.0)	2.774	0.939–8.192	0.065
Coronary heart disease	7(4.8)	2.343	0.987–5.560	0.054
Previous stroke/TIA	8(2.4)	0.985	0.434–2.234	0.971
Smoking	9(2.3)	0.900	0.408–1.983	0.794
Drinking	6(2.6)	1.093	0.442–2.706	0.847
High A^2^DS^2^ score group	18(7.8)	7.014	3.329–14.779	<0.001

TIA indicates transient ischemic attack; NIHSS, National Institutes of Health Stroke Scale.

**Table 4:** The predictive value of the A^2^DS^2^ Score for SAP and in-hospital mortality after AIS.

	SAP		In-hospital death	
Variable	Unadjusted OR (95%CI)	Adjusted OR (95%CI)	Unadjusted OR (95%CI)	Adjusted OR (95%CI)
the A^2^DS^2^ score	1.779* (1.583–2.000)	1.759* (1.560–1.984)	1.681* (1.401–2.018)	1.753* (1.444–2.129)
the A^2^DS^2^ score group				
Low score group	Ref	Ref	Ref	Ref
High score group	9.679* (6.122–15.302)	8.888* (5.552–14.229)	7.014* (3.329–14.779)	7.833* (3.580–17.137)

SAP indicates stroke-associated pneumonia. AIS, acute ischemic stroke.

* *P*<0.001

^a^ Multivariable logistic regression adjusted for history of dyslipidemia, hypertension, diabetes, stroke or transient ischemic attack, coronary heart disease, smoke and drink.

## Discussion

SAP is a common medical complication and an important risk factor for mortality after stroke, with rates reported between 5.6% and 37.98%[1–11]. In our study, the incidence rate of SAP 7.3% was lower than some other studies[20,22], which may be due to two reasons. Firstly, the NIHSS score median in our study was 3 (IQR 2–6), while Li’s [22] and Ji’s studies[20] identified median scores of 4 (IQR 2–6) and 5 (IQR 2–9). Secondly, dysphagia screening was routinely performed among stroke patients in our department which was proved to be effective in prevention of SAP[27].The present study found that 39 (43.3%) SAP was early-onset pneumonia, which is in line with other studies [1,3], indicating that early-onset pneumonia developed in 43%-79% of SAP patients. This indicates that it is important to identify patients with a high risk of SAP based on routinely collected data immediately upon admission.

The results of our study showed that high A^2^DS^2^ score group (5–10) had higher risk of SAP than low A^2^DS^2^ score group. According to the results of our study, stroke patients whose A^2^DS^2^ score was high should get more attention on SAP and might get early prevention or treatment than patients with low A^2^DS^2^ score. Given that dysphagia is one of the most important risk factor for SAP[17–20,22], we are currently running a prospective study for the prevention of SAP through dynamic dysphagia assessment in the AIS patients with a high risk of SAP.

Our study also revealed that the A^2^DS^2^ score was an important predictor of in-hospital death. Prior study proved clinicians with expertise in stroke performed poorly compared to a validated tool in predicting the outcomes of patients with an acute ischemic stroke. Use of a prognostic tool may be superior for decision-making following an acute ischemic stroke[28]. Several prognostic scores (e.g. Smith’s risk score[29], Saposnik’s iscore [30] and Myint’s SOAR score[31]) had been developed to predict mortality in Acute Stroke. However, Smith’s risk score and Saposnik’s iscore both consisted of 12 components and were very complex; the positive predictive values of the SOAR score were relatively low. Given that most relevant clinical decisions are usually made in the first few days after admission, it’s important to predict in-hospital death as soon as possible after hospital admission. The A^2^DS^2^ score was a simple and convenient scoring system and our study indicated that the A^2^DS^2^ score was an important predictor of in-hospital death. Patients in the high score group had a higher risk of in-hospital death compared with patients in the low score group (OR = 7.833; 95% CI, 3.580–17.137).

This study has several strengths. First, our findings demonstrate that the A^2^DS^2^ score is a strong predictor for SAP and in-hospital death when the A^2^DS^2^ score was dichotomized into low and high score groups. In this case, it is easy and efficient to use the A^2^DS^2^ score in clinical practice or clinical trial. Second, to our knowledge, this is the first study to use the A^2^DS^2^ score as a predictor of in-hospital death for AIS patients and demonstrates that the A^2^DS^2^ score is an important predictor of in-hospital death. Third, though the Centers for Disease Control and Prevention criteria for the diagnosis of pneumonia were not applied, pneumonia in our study is comparable to that of other large studies[19,20], making large quantities of misdiagnoses of SAP unlikely. Moreover, the present study documents the exact date of SAP onset, which demonstrates that the incidence of early-onset pneumonia was high and it’s important to predict SAP as soon as possible after admission.

Our study also had some limitations. First, our study is a retrospective study from a single center, which could have impacted selection bias. Second, because this is an observational study, we cannot rule out the possibility that the results might be affected by some unmeasured confounders such as the use of angiotensin-converting enzyme inhibitors or angiotensin receptor blockers[32], β-Blocker[33] and metoclopramide[34]. Third, in our study, we did not analyze the association between mechanical ventilation and outcome. Ventilator-associated pneumonia is assumed to be caused by mechanical ventilation itself and other risk factors that substantially differ from those identified for the development of SAP[35]. Also, in the process of developing the A^2^DS^2^ score, restricting the analysis to patients not ventilated did not change any of the observed association substantially [19].

This study was a retrospective study, thus, there is a need for further prospective validation. We believe that the A^2^DS^2^ score is a useful tool for predicting SAP and death during hospitalization, which will aid clinical decision-making for patients who have AIS.

## Conclusions

The A^2^DS^2^ score was a strong predictor for SAP and in-hospital death in Chinese AIS patients. The A^2^DS^2^ score might be a useful tool for identifying patients with a high risk of SAP and death during hospitalization.

## Supporting Information

S1 Text PLOS One Clinical Studies Checklist. (DOCX)

S2 Text STROBE checklist v4 combined PlosMedicine. (DOCX)
