# Supplementary material for: The A2DS2 Score as a Predictor of Pneumonia and In-Hospital Death after Acute Ischemic Stroke in Chinese Populations
Source: PLoS One. 2016 Mar 7;11(3):e0150298. doi: 10.1371/journal.pone.0150298 (PMC4780726; doi:10.1371/journal.pone.0150298)
Supplement: S2 Text — (DOCX) [file pone.0150298.s002.docx]

STROBE Statement—checklist of items that should be included in reports of observational studies

|  | Item No. | Recommendation | Page  No. | Relevant text from manuscript |
| --- | --- | --- | --- | --- |
| **Title and abstract** | 1 | (*a*) Indicate the study’s design with a commonly used term in the title or the abstract | 1 | This is a retrospective study. |
|  |  | (*b*) Provide in the abstract an informative and balanced summary of what was done and what was found | 2-3 | We sought to identify the predictive value of the A^2^DS^2^ score for SAP and in-hospital death after acute ischemic stroke, and found that the A^2^DS^2^ score could predict SAP and in-hospital death of Chinese acute ischemic stroke patients |
| Introduction | | | |  |
| Background/rationale | 2 | Explain the scientific background and rationale for the investigation being reported | 3-4 | Stroke-associated pneumonia (SAP) is a common medical complication after stroke. To effectively evaluate the risk of SAP, several scales have been developed, but the A^2^DS^2^ score is the simplest scale. The previous study used the A^2^DS^2^ score as a continuous variable to predict SAP. However, in the clinical practice, the dichotomized cutoff point was more convenient. |
| Objectives | 3 | State specific objectives, including any prespecified hypotheses | 4 | The objective of our study is to identify the predictive value of A^2^DS^2^ score as dichotomized variable for SAP, furthermore, to identify whether it is predictive in death during hospitalization among Chinese ischemic stroke population. |
| Methods | | | |  |
| Study design | 4 | Present key elements of study design early in the paper | 5-6 | Our study was approved by the ethic committee (2008 GL-37) of 2nd Affiliated Hospital of Guangzhou University of Chinese Medicine. Our study was a retrospective study and all information was pulled out through the electronic system. |
| Setting | 5 | Describe the setting, locations, and relevant dates, including periods of recruitment, exposure, follow-up, and data collection | 4 | Subjects were acute ischemic stroke (AIS) patients admitted to the department of neurology in Guangdong Provincial Hospital of Chinese Medicine between August 2005 and July 2008. |
| Participants | 6 | (*a*) *Cohort study*—Give the eligibility criteria, and the sources and methods of selection of participants. Describe methods of follow-up  *Case-control study*—Give the eligibility criteria, and the sources and methods of case ascertainment and control selection. Give the rationale for the choice of cases and controls  *Cross-sectional study*—Give the eligibility criteria, and the sources and methods of selection of participants | 4 | Inclusion criteria were: (1) ischemic stroke verified by Computerized Tomography (CT) or Magnetic Resonance Imaging (MRI); and (2) time from symptom onset within7 days. Patients were excluded if any of the components of the A^2^DS^2^ score were not available. |
|  |  | (*b*) *Cohort study*—For matched studies, give matching criteria and number of exposed and unexposed  *Case-control study*—For matched studies, give matching criteria and the number of controls per case |  |  |
| Variables | 7 | Clearly define all outcomes, exposures, predictors, potential confounders, and effect modifiers. Give diagnostic criteria, if applicable | 5-6 | Variables include:(1) demographics (i.e., age and sex); stroke risk factors (i.e., hypertension, diabetes mellitus, dyslipidemia, and atrial fibrillation, including a history of atrial fibrillation or documentation of atrial fibrillation at admission, coronary heart disease (CHD), stroke and TIA history, smoking, and drinking); (2) stroke severity at admission as assessed by the National Institute of Health Stroke Scale score (NIHSS); dysphagia ( Kubota’s water swallow test Grade III or higher); (3)outcomes including pneumonia and death during hospitalization. SAP was diagnosed by the treating physician according to Mann’s diagnostic criteria for pneumonia. The A^2^DS^2^ score was calculated (Age≥75 years=1, Atrial fibrillation=1, Dysphagia=2, male Sex=1, stroke Severity, National Institutes of Health Stroke Scale 0-4=0, 5-15=3, ≥16=5) and was dichotomized into low (0-4) and high (5-10) score groups. |
| Data sources/ measurement | 8* | For each variable of interest, give sources of data and details of methods of assessment (measurement). Describe comparability of assessment methods if there is more than one group | *5-6* | stroke severity at admission as assessed by the National Institute of Health Stroke Scale score (NIHSS); dysphagia ( Kubota’s water swallow test Grade III or higher); SAP was diagnosed by the treating physician according to Mann’s diagnostic criteria for pneumonia |
| Bias | 9 | Describe any efforts to address potential sources of bias | 6 | We enlarge the sample size. The investigators verified the AIS diagnosis according to the CT/MRI results. |
| Study size | 10 | Explain how the study size was arrived at | 5 | In logistic regression analysis, accurate estimation of the discriminant function parameters demands sample size of minimum 20 cases for each predictor variable. |

Continued on next page

| Quantitative variables | 11 | Explain how quantitative variables were handled in the analyses. If applicable, describe which groupings were chosen and why | 6 | The data were presented as the mean ±standard deviation (SD), the median with interquartile ranges (IQR), or frequencies with percentages, as appropriate. |
| --- | --- | --- | --- | --- |
| Statistical methods | 12 | (*a*) Describe all statistical methods, including those used to control for confounding | 6 | Student’s t- or Kruskal-Wallis tests; Chi-square tests; univariate and multivariate logistic regression analyses |
|  |  | (*b*) Describe any methods used to examine subgroups and interactions |  |  |
|  |  | (*c*) Explain how missing data were addressed | 4 | Patients were excluded if any of the components of the A^2^DS^2^ score were not available. |
|  |  | (*d*) *Cohort study*—If applicable, explain how loss to follow-up was addressed  *Case-control study*—If applicable, explain how matching of cases and controls was addressed  *Cross-sectional study*—If applicable, describe analytical methods taking account of sampling strategy |  |  |
|  |  | (*e*) Describe any sensitivity analyses | No | In our study, we didn’t use any sensitivity analyses. |
| Results | | | | |
| Participants | 13* | (a) Report numbers of individuals at each stage of study—eg numbers potentially eligible, examined for eligibility, confirmed eligible, included in the study, completing follow-up, and analysed | 7 | Records of 1504 patients with the diagnosis of “ischemic stroke” or “cerebral infarction” were extracted from the electronic medical system. 265 patients were excluded because time from stroke onset was more than 7 days. At last, 1239 patients were analysed. |
|  |  | (b) Give reasons for non-participation at each stage | No | Because the outcome was SAP and in-hospital mortality, and the stage was from admission to discharge. |
|  |  | (c) Consider use of a flow diagram | 7 | Study population flowchart |
| Descriptive data | 14* | (a) Give characteristics of study participants (eg demographic, clinical, social) and information on exposures and potential confounders | 7 | Table 1.The patients’ mean age was 69.056±11.662, and 732 patients (59.1%) were men.122 patients (9.8%) had atrial fibrillation, 204 patients (16.5%) had dysphagia symptoms, and the NIHSS median score was 3 (IQR 2-6). The median A^2^DS^2^ score was 2(IQR1-4).1008 patients (81.4%) were in the low A^2^DS^2^ score group and 231(18.6%) were in the high A^2^DS^2^ score groups. |
|  |  | (b) Indicate number of participants with missing data for each variable of interest | 7 | None data for variable of interest was missing. |
|  |  | (c) *Cohort study*—Summarise follow-up time (eg, average and total amount) |  |  |
| Outcome data | 15* | *Cohort study*—Report numbers of outcome events or summary measures over time |  |  |
|  |  | *Case-control study—*Report numbers in each exposure category, or summary measures of exposure | *7-8* | Table1-3. The overall incidence rate of SAP and in-hospital mortality was 7.3% and 2.4%, respectively. Age, atrial fibrillation, dysphagia, admission NIHSS score, history of CHD, and high A^2^DS^2^ score were risk factors for SAP. Dyslipidemia, atrial fibrillation, dysphagia, NIHSS at admission and the A^2^DS^2^ score were risk factors for death during hospitalization. |
|  |  | *Cross-sectional study—*Report numbers of outcome events or summary measures |  |  |
| Main results | 16 | (*a*) Give unadjusted estimates and, if applicable, confounder-adjusted estimates and their precision (eg, 95% confidence interval). Make clear which confounders were adjusted for and why they were included | 8-10 | Table2-4. The multivariate analysis proved that higher A^2^DS^2^ score was associated with higher risk of SAP (OR=1.759; 95%CI, 1.560-1.984) even after adjustment for traditional stroke risk factors. Subjects in high A^2^DS^2^ score group had higher risk for SAP (OR=8.888; 95%CI, 5.552-14.229) compared to low A^2^DS^2^ score group. Multivariate logistic regression showed that the A^2^DS^2^ score’s OR for in-hospital mortality was 1.753 (95%CI, 1.444-2.129) after adjustment for vascular risk factors. Patients in the high score group had a higher risk of in-hospital death than patients in the low score group (adjusted OR= 7.833; 95% CI, 3.580-17.137). |
|  |  | (*b*) Report category boundaries when continuous variables were categorized | 8-10 | Table1 Age were categorized as ≥75 or <75 and the A^2^DS^2^ score was dichotomized into low score group (0-4) and high score group (5-10). |
|  |  | (*c*) If relevant, consider translating estimates of relative risk into absolute risk for a meaningful time period |  |  |

Continued on next page

| Other analyses | 17 | Report other analyses done—eg analyses of subgroups and interactions, and sensitivity analyses |  |  |
| --- | --- | --- | --- | --- |
| Discussion | | | | |
| Key results | 18 | Summarise key results with reference to study objectives | 10-12 | The overall incidence rates of SAP and in-hospital mortality after acute ischemic stroke were 7.3% and 2.4%, respectively. The incidence rate of SAP in low and high A^2^DS^2^ score groups was separately 3.3% and 24.7% (P<0.001). During hospitalization, 1.2% patients in low score group and 7.8% patients in high score group died (P<0.001). Multivariate regression demonstrated that patients in high score group had a higher risk of SAP (OR= 8.888, 95%CI: 5.552-14.229) and death (OR=7.833, 95%CI: 3.580-17.137) than patients in low score group. |
| Limitations | 19 | Discuss limitations of the study, taking into account sources of potential bias or imprecision. Discuss both direction and magnitude of any potential bias | 12 | Our study is a retrospective study and we cannot rule out the possibility that the results might be affected by some unmeasured confounders. |
| Interpretation | 20 | Give a cautious overall interpretation of results considering objectives, limitations, multiplicity of analyses, results from similar studies, and other relevant evidence | 13 | The A^2^DS^2^ score could predict SAP and death during hospitalization in Chinese AIS patients. |
| Generalisability | 21 | Discuss the generalisability (external validity) of the study results | 13 | The A^2^DS^2^ score could and should be used in clinical practice as a useful tool for identifying patients with a high risk of SAP and death during hospitalization. |
| Other information | |  | | |
| Funding | 22 | Give the source of funding and the role of the funders for the present study and, if applicable, for the original study on which the present article is based | 13 | This project was supported by the Scientific Research Project of Public Welfare Industry, State Administration of Traditional Chinese Medicine of P. R. of China  (No. 200707004); and the Finance Department of Guangdong Province (No. [2006] 143). The funders had no role in the study design, data collection and analysis, decision to publish, or manuscript preparation. |

*Give information separately for cases and controls in case-control studies and, if applicable, for exposed and unexposed groups in cohort and cross-sectional studies.

**Note:** An Explanation and Elaboration article discusses each checklist item and gives methodological background and published examples of transparent reporting. The STROBE checklist is best used in conjunction with this article (freely available on the Web sites of PLoS Medicine at http://www.plosmedicine.org/, Annals of Internal Medicine at http://www.annals.org/, and Epidemiology at http://www.epidem.com/). Information on the STROBE Initiative is available at www.strobe-statement.org.
